# Glasgow Royal Infirmary

**Published:** 1833-08

**Authors:** 


### REPORTS

### FROM HOSPITALS AND MEDICAL INSTITUTIONS.

### GLASGOW ROYAL INFIRMARY.

*Cases of Urinary Abscess.* [From MACFARLANE's *Surgical Reports.*]

CASE I. *Stricture of the Urethra, giving rise to Urinary Abscess and Extravasation: Cure.*

R. C., æt. thirty-five, admitted 23d May, 1831. Had frequent calls to void urine, which was passed after painful and long-continued straining, in a twisted stream not larger than a thread. These symptoms commenced five years before, after a gonorrhœa, which was cured by stimulating injections. On the introduction of a small catgut bougie, a stricture was discovered about three inches from the orifice of the urethra, behind which a good deal of thickening and irregularity of the canal was felt for more than two inches. There was also a second stricture behind the bulb, through which the smallest instrument could not be passed. The prostate gland was enlarged.

On the evening of the day on which he was admitted, and before I had an opportunity of examining his urethra, he had a smart rigor, followed by the usual febrile excitement. On the following day, I found him labouring under acute peritonitis; the abdomen was tumid and exquisitely painful; there was incessant vomiting, urgent thirst and constipation; the countenance was anxious, and the pulse small, sharp, and one hundred and twenty in the minute. He was put under the antiphlogistic treatment, both general and local; but for several days the symptoms continued alarming, and did not subside until ptyalism was excited by repeated doses of calomel and opium.

On the 1st of June, he complained of increased pain and difficulty in micturition; and his urine was for the first time tinged with blood. A small, hard, circumscribed tumor was discovered in the perinæum, immediately behind the posterior edge of the scrotum, where the irregularity leading from the first stricture terminated. As it seemed to threaten the formation of an abscess, leeches were applied, followed by fomentations, an anodyne enema, &c.; but although it got larger during the two following days, it did not fluctuate. At this time I hesitated as to the propriety of opening the tumor, particularly when I found that, by pressing it, a few drops of pus escaped from the urethra. It appeared probable that a portion of the canal connected with the external tumor had ulcerated, and that the pus was contained in a circumscribed cavity in the corresponding part of the corpus spongiosum. Had I been satisfied that this supposition was correct, I would not have hesitated to make a free incision into the part, from a conviction, that had the urethra been actually perforated by ulceration, the urine, which might have been prevented for a few days from escaping into the surrounding parts by the lining of lymph forming the walls of the abscess, must soon have destroyed this barrier, and become extensively diffused into the cellular texture of the perinæum and scrotum. But as the perineal tumor was small, hard, and circumscribed, and as it was possible that the pus, which could be pressed from it along the penis, might be furnished from the surface of the dilated urethra, immediately behind the stricture without the existence of a breach in the canal, I determined to watch the case narrowly, and to delay making an incision until the appearances were less equivocal. I have seen more than one case, where, by gradually pressing the pus along the urethra, and preventing its accumulation at the affected part, the tumor has disappeared, and an external opening been rendered unnecessary. I would therefore be extremely cautious in making incisions into the perinæum, unless extravasation of urine had actually occurred, or was unequivocally impending, especially in the unhealthy and broken down individuals who are so generally the subjects of this disease.

On the evening of the 3d, the tumor in the perinæum began to increase, and to lose its circumscribed form; and, before the hour of visit on the 4th, the tumefaction had extended along the scrotum, penis, and groins, to near the umbilicus: the skin was red and hot, and there was less urine passed by the penis than formerly. It was evident that the abscess had given way, and that the urine was escaping by a preternatural opening in the urethra. The patient was therefore placed on a table, and secured as for lithotomy, a catheter was passed until its point rested on the stricture, and an incision two inches in length made into the tumor, giving exit to pus and urine, and showing that the walls of the abscess were thick, and lined by a dense layer of lymph. One or two gentle attempts were made to pass a small catheter into the bladder, both by the penis and wound, but, from the soft and sloughy state of the parts, this was impracticable. It was to be expected, however, that the free opening which was made would permit the urine to escape, and put an end to farther extravasation. A pledget of oiled lint was introduced between the edges of the wound; the tumefied integuments were freely scarified; warm fomentations applied; and he was ordered a grain and a half of opium at bed-time.

5th. Is much better, has passed a quiet night, and countenance is less anxious; swelling and redness of integuments greatly diminished; voids his urine freely through the wound; pulse one hundred and sixteen, soft; tongue white, but moist.

For several days the constitutional symptoms continued moderate, and the integuments of the scrotum and penis regained their natural appearance without sloughing taking place; but there was a good deal of hardness and erysipelatous redness of the abdominal integuments, from the symphisis pubis to near the umbilicus. On the 10th, fluctuation was felt; two openings were made, and several ounces of fetid pus, with some shreds of dead cellular substance, were discharged. This abscess was found to communicate with the opening in the perinæum, showing that there was a considerable destruction of the cellular texture between the penis and pubes. Ordered full diet, and six ounces of wine daily.

The symptoms continued favourable; the discharge from the different openings was moderate; their edges were florid and granulating; and his health was improving. On the evening of the 23d he had a smart rigor, and at the visit on the 24th he complained of pain, on pressure, in the lower part of abdomen; the pulse was one hundred and thirty, sharp; the tongue dry and furred; the skin hot, but moist; and the respiration hurried; the half of the wound in the perinæum had closed, but the urine continued to pass through it in a full and free stream.—Cluniluvium stat. Ol. Ricini.

On the 25th, the febrile symptoms and abdominal pain had almost subsided; and in a few days I again attempted to pass a catheter into the bladder, but without success. The urethra, from about the middle of the scrotum to the bulb, was so soft and ragged, that the instrument, by the slightest force, could have been passed in any direction: I therefore ceased to attempt its introduction, until the parts had become firmer and more consolidated.

On the 2d July, the openings in perinæo and above pubes were nearly healed; and the whole urine was voided by the penis in a small irregular stream. On the 20th, the parts were completely cicatrized, and for the first time a small catgut bougie was passed through the stricture into the bladder. This was repeated daily, and retained for nearly an hour, the size of the instrument being gradually increased, until the largest could be easily introduced. By this treatment, the stream of urine increased; the stricture was destroyed; the irregularity of the canal removed; and he was dismissed, cured, on the 29th of September.

The necessity for prompt and active treatment, and the great benefit of free and early incisions in this class of diseases, are now well known and acknowledged. The object of the treatment consists in laying freely open the perineal tumor, so as to promote the egress of the urine, and prevent its extensive diffusion in the cellular texture. When extravasation occurs, it extends with great rapidity, and the vitality of the parts is speedily destroyed. I have, however, succeeded in two cases, where the scrotum and penis were red and swollen, by early and free incisions, followed by warm anodyne fomentations, not only in arresting the progress of the extravasation, but also in preventing that destructive sloughing of the parts which is so generally and rapidly produced. The extent to which the urine has spread, can in general be ascertained by the dusky redness of the integuments, which is always present, and which sometimes makes its appearance in a very few minutes after the extravasation has taken place into the subjacent cellular texture. The history and peculiar character of this disease are in general so obviously and unequivocally marked, as seldom to render its recognition difficult. I have met with two cases, however, in which it was mistaken for other diseases by well-informed surgeons; and, in one of these, the neglect of the proper treatment proved fatal.

Case II. *Urinary Abscess and Extravasation mistaken for Erysipelas: Death.*

I was requested by a surgeon in town to visit an old soldier, who had long laboured under stricture of the urethra. Eight days previously, a small painful tumor began to form in the perinæum, which gradually increased; and was followed, in four days, by a sudden swelling of the scrotum and penis, which extended rapidly to the groins, upper part of the thighs, and abdomen, and was speedily productive of redness of the integuments; which in a few hours assumed a violet colour. The urine continuing to dribble from the penis, it was supposed that the disease was erysipelas, and that the external swelling had no connexion with the urethra. I made several free incisions into the affected parts, which were gangrenous, and gave exit to a large quantity of fetid urine mixed with pus. Through one of these openings, in the situation of the original tumor in the perinæum, I succeeded in passing an elastic catheter into the bladder, and drawing off two pounds of urine. From the extent of the extravasation, the dissipated habits of the patient, who was a dram-drinker, and had been long in a warm climate, and the great typhoid depression which existed, but little benefit followed this treatment. He did not survive more than twelve hours.

On dissection, besides the great destruction of the cellular texture and integuments externally, it was found that the urine was extensively extravasated into the cellular substance of the pelvis, having passed along the crura of the penis, and under the symphisis pubis, so as to form a communication with the sloughy cavity on the surface of the abdominal muscles.

Should the usual symptoms of inflammatory fever have attended the formation and advancement of the tumor in the perinæum, we find that these change their type, and become distinctly typhoid, so soon as the urine has begun to spread into the adjoining cellular substance. I have also observed, that these in their turn disappear, the countenance losing its sunk and haggard appearance, the tongue its red edges and furred surface, the pulse its irritable and wiry beat, and the skin its coldness, whenever the further extravasation of urine is prevented by a free incision into the perinæum, and the cellular texture is unloaded by numerous scarifications.

